# Supplementary material for: Causal Relationship between Aging and Anorexia Nervosa: A White-Matter-Microstructure-Mediated Mendelian Randomization Analysis
Source: Biomedicines. 2024 Aug 16;12(8):1874. doi: 10.3390/biomedicines12081874 (PMC11351342; doi:10.3390/biomedicines12081874)
Supplement: Supplementary file 1 [file biomedicines-12-01874-s001.zip › Supplementary Figure/Figure S8/Leaveoneout plot of MR analysis for TL to ubm-b-2051.pdf]

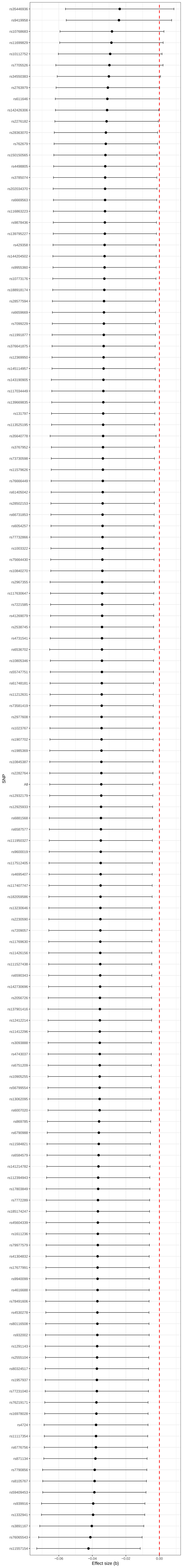
rs35446936
rs9419958
rs10768683
rs11699829
rs10112752
rs7705526
rs34550383
rs2763979
rs611646
rs142426306
rs2276182
rs28363070
rs762679
rs150150565
rs4498805
rs3785074
rs202034370
rs6669563
rs116863223
rs9878436
rs139795227
rs429358
rs144204502
rs9955360
rs10773176
rs188918174
rs28577594
rs6659669
rs7099229
rs11991877
rs376641875
rs12369950
rs145114957
rs143190905
rs117034449
rs139669835
rs131797
rs113525195
rs35640778
rs3767952
rs73730598
rs11579626
rs76666449
rs61405042
rs28502153
rs66731853
rs6054257
rs77732866
rs1003322
rs75664430
rs10840270
rs2967355
rs117630647
rs7221585
rs41269079
rs2538745
rs4731541
rs6536702
rs10805346
rs55747751
rs61748181
rs11212631
rs73581419
rs2977608
rs1023767
rs1907702
rs1985369
rs10845387
rs2282764
All
rs12932179
rs12925933
rs6881568
rs6587577
rs111950327
rs9600019
rs117512405
rs4695407
rs117407747
rs182059586
rs13230646
rs2230590
rs7209057
rs11769630
rs11426156
rs111527438
rs6590343
rs142730696
rs2056726
rs137901416
rs12412214
rs11412296
rs3093888
rs4743037
rs6751209
rs10905255
rs56799554
rs13062095
rs6007020
rs869785
rs6790988
rs11584821
rs6584579
rs141214782
rs112394943
rs17803849
rs7772289
rs185174247
rs45604339
rs1611236
rs79977579
rs41304832
rs17677991
rs9940099
rs4616688
rs78491606
rs4530278
rs80116508
rs932002
rs1291143
rs2555104
rs80324517
rs1957937
rs77231040
rs76219171
rs16978028
rs4724
rs11117354
rs6776756
rs871134
rs7790856
rs8105767
rs59409453
rs939916
rs1332941
rs3891167
rs76065543
rs11557154
SNP
−0.06
−0.04
−0.02
0.00
Effect size (b)
